# An efflux pump in genomic island GI-M202a mediates the transfer of polymyxin B resistance in *Pandoraea pnomenusa* M202

**DOI:** 10.1007/s10123-023-00384-8

**Published:** 2023-06-15

**Authors:** Wenhui Gao, Congcong Li, Fengtian Wang, Yilin Yang, Lu Zhang, Zhongxue Wang, Xi Chen, Meixia Tan, Guangxiang Cao, Gongli Zong

**Affiliations:** 1 First Affiliated Hospital of Shandong First Medical University, Biomedical Sciences College & Shandong Medicinal Biotechnology Centre, Shandong First Medical University & Shandong Academy of Medical Sciences, Ji’nan, 250117 China; 2 NHC Key Laboratory of Biotechnology Drugs (Shandong Academy of Medical Sciences), Ji’nan, 250117 Shandong China; 3 Shandong Quancheng Test & Technology Limited Company, Ji’nan, 250101 China; 4 Jinan Municipal Minzu Hospital, Ji’nan, 250012 China

**Keywords:** *Pandoraea pnomenusa*, Polymyxin B resistance, Genomic island, MFS transporter

## Abstract

**Background:**

Polymyxin B is considered a last-line therapeutic option against multidrug-resistant gram-negative bacteria, especially in COVID-19 coinfections or other serious infections. However, the risk of antimicrobial resistance and its spread to the environment should be brought to the forefront.

**Methods:**

*Pandoraea pnomenusa* M202 was isolated under selection with 8 mg/L polymyxin B from hospital sewage and then was sequenced by the PacBio RS II and Illumina HiSeq 4000 platforms. Mating experiments were performed to evaluate the transfer of the major facilitator superfamily (MFS) transporter in genomic islands (GIs) to *Escherichia coli* 25DN. The recombinant *E. coli* strain Mrc-3 harboring MFS transporter encoding gene FKQ53_RS21695 was also constructed. The influence of efflux pump inhibitors (EPIs) on MICs was determined. The mechanism of polymyxin B excretion mediated by FKQ53_RS21695 was investigated by Discovery Studio 2.0 based on homology modeling.

**Results:**

The MIC of polymyxin B for the multidrug-resistant bacterial strain *P. pnomenusa* M202, isolated from hospital sewage, was 96 mg/L. GI-M202a, harboring an MFS transporter-encoding gene and conjugative transfer protein-encoding genes of the type IV secretion system, was identified in *P. pnomenusa* M202. The mating experiment between M202 and *E. coli* 25DN reflected the transferability of polymyxin B resistance via GI-M202a. EPI and heterogeneous expression assays also suggested that the MFS transporter gene FKQ53_RS21695 in GI-M202a was responsible for polymyxin B resistance. Molecular docking revealed that the polymyxin B fatty acyl group inserts into the hydrophobic region of the transmembrane core with Pi-alkyl and unfavorable bump interactions, and then polymyxin B rotates around Tyr43 to externally display the peptide group during the efflux process, accompanied by an inward-to-outward conformational change in the MFS transporter. Additionally, verapamil and CCCP exhibited significant inhibition via competition for binding sites.

**Conclusions:**

These findings demonstrated that GI-M202a along with the MFS transporter FKQ53_RS21695 in *P. pnomenusa* M202 could mediate the transmission of polymyxin B resistance.

**Supplementary Information:**

The online version contains supplementary material available at 10.1007/s10123-023-00384-8.

## Introduction

The relatively rapid global spread of and rise in COVID-19 cases prompted the WHO to declare the disease a pandemic on 11 March 2020 (O'Toole 2021). Studies have indicated that antibiotics are prescribed frequently to patients with COVID-19, largely due to suspected bacterial coinfections (Langford et al. 2021). Despite frequent antibiotic prescription, the prevalence of bacterial coinfection and secondary infection in patients hospitalized with COVID-19 is 3.5% and 14.3%, respectively (Langford et al. 2020). Another major concern regarding hospitalized patients with COVID-19 is bacterial superinfections, especially in the intensive care setting and in patients using invasive devices, as well as in outbreaks of multidrug-resistant bacteria resulting from poorer adherence to infection control practices (Perez et al. 2020; Rawson et al. 2020; Sharifipour et al. 2020). The high frequency of antibiotic prescription highlights the potential for significant antibiotic overuse in these patients (Langford et al. 2021) and antimicrobial resistance (AMR) as a potential consequence. The increase in unnecessary antimicrobial use will potentially enhance the future risk of AMR by driving the selection of multidrug-resistant (MDR) organisms (Rawson et al. 2020). Multiple studies worldwide have reported an unexpectedly high incidence of infections due to methicillin-resistant *Staphylococcus aureus* (MRSA), methicillin-resistant *Acinetobacter baumannii* (MRAB), carbapenem-resistant *A. baumannii* (CRAB), and carbapenem-resistant Enterobacteriaceae (CRE) among COVID-19 patients admitted to the intensive care unit (Segala et al. 2021).

Polymyxins are amphipathic lipopeptide molecules with strong bactericidal activity against a range of gram-negative bacteria. Polymyxin antibiotics, polymyxin B in particular, are increasingly being used as last-line therapeutic options against MDR bacterial infections (Moffatt et al. 2019). Recently, polymyxin B was reported as an effective drug for use in COVID-19 patients with bacterial coinfections (Elmorsy et al. 2022). However, with the increased use of polymyxin B, resistant strains are emerging at an alarming rate. Thus, the repercussions of COVID-19 with bacterial coinfections on AMR are of great concern due to elevated antibiotic use in patients infected with COVID-19 (Hughes et al. 2020). Opportunistic pathogens can also cause superinfections, especially in combination with viral respiratory tract infections in hospitalized patients (Sharifipour et al. 2020). MDR organisms have emerged not only in the hospital environment but also in community settings, suggesting that reservoirs of antibiotic-resistant bacteria are present around hospitals (Youlden et al. 2022). *Pandoraea* species is a newly emerging multidrug-resistant pathogen usually isolated from a variety of clinical samples (See-Too et al. 2019; Gawalkar et al. 2021; Singh et al. 2021; Cubides-Diaz et al. 2022; Ramos Oliveira et al. 2023) with antibiotic resistance (Schneider et al. 2006). In this study, we isolated the MDR gram-negative strain *P. pnomenusa* M202 from hospital sewage. M202 could spread polymyxin B resistance via a newly identified MFS transporter present on a genomic island (GI). These results provide new insights into the mechanisms of dissemination of polymyxin B resistance and raise concerns regarding antibiotic therapy for bacterial infections.

## Materials and methods

### Isolation and identification of the multidrug-resistant *P. pnomenusa* M202

Hospital sewage was obtained from the wastewater treatment facility in Shandong Province, China. The sewage samples were diluted and spread onto Luria–Bertani (LB) agar plates (0.5% yeast extract, 1% tryptone, 1% sodium chloride, 2% agar) containing 8 mg/L polymyxin B (Sigma Co. Shanghai, China) and then incubated at 28 °C for 24 h. All colonies with different phenotypes on plates were selected and cultivated three consecutive times on LB agar medium with antibiotics to obtain single colonies. After further purification, one single colony, named M202, was selected and grown as a pure culture. For multidrug resistance analysis, antimicrobial susceptibility tests were performed to determine the MICs for 9 antibiotics, including ampicillin, cefixime, tetracycline, ciprofloxacin, florfenicol, amikacin, polymyxin B, sulfamethoxazole, and meropenem, based on the breakpoints defined by the Clinical and Laboratory Standards Institute (CLSI 2020).

Genomic DNA of *P. pnomenusa* M202 was extracted using the Genomic DNA Purification Kit (Promega, WI, USA) and analyzed using a NanoDrop UV–Vis spectrophotometer (Thermo Scientific, MA, USA). 16S rRNA genes were amplified with the universal primers 27F (5′-AGAGTTTGATCCTGGCTCAG-3′) and 1492R (5′-TACCTTGTTACGACTT-3′) and sequenced. Similarity analyses of the 16S rRNA sequences were conducted for preliminary identification using BLASTn (https://blast.ncbi.nlm.nih.gov/Blast.cgi). A phylogenetic tree of M202 was produced by using neighbor-joining algorithms in Molecular Evolutionary Genetics Analysis 7 (MEGA 7) software based on the genome sequencing results of the 16S rDNA sequence (Jeong et al. 2016).

### Whole-genome sequencing, annotation, and analysis

*P. pnomenusa* M202 genome was sequenced by the Nanopore and BGISEQ-500 platforms at BGI Co., Ltd. (Wuhan, China). The M202 genome was assembled by glimmer3 (http://www.cbcb.umd.edu/software/glimmer/) with hidden Markov models (Tatusova et al. 2016). Genome annotation was performed using the Prokaryotic Genome Annotation Pipeline of NCBI (http://ncbi.nlm.nih.gov/genome/annotation_prok/). The genome alignment was performed by the PATRIC server (Brettin et al. 2015). GIs were predicted by IslandViewer 4 (Bertelli et al. 2017). The conjugation system was identified by the PATRIC server (Brettin et al. 2015) and oriTfinder (Li et al. 2018).

### Analysis of antibiotic resistance genes

Antibiotic resistance genes (ARGs) were analyzed by RAST and BLASTp based on the core dataset in the Antibiotic Resistance Genes Database (ARDB) (Liu and Pop 2009). Multisequence comparison was carried out by Clustal Omega (Madeira et al. 2019) and ESPript (Robert and Gouet 2014).

### Mating experiments

Broth-based mating experiments were carried out using M202 as the donor and *Escherichia coli* 25DN as the recipient as described previously (Zhang et al. 2020; Zong et al. 2020). Transconjugants were screened when 8 mg/L polymyxin B was used for selection. To determine whether GI-M202a was transferred to transconjugants, the transconjugant clone M202-TC1 was analyzed by PCR with three primer pairs (Table S1), including V1-F/R (for the helix-turn-helix domain-containing protein gene, FKQ53_RS21685), V2-F/R (for the MFS gene, FKQ53_RS21695), and V3-F/R (for the RidA family protein gene, FKQ53_RS21700). The MICs of antibiotics for the transconjugants were determined as described above.

### Construction of the FKQ53_RS21695 recombinant *E. coli* strain

The MFS transporter-encoding gene FKQ53_RS21695 in GI-M202a was amplified using the primer pair MRC-F/R (with *Xba* I at the 5′-end and *Hind* III at the 3′-end) and M202 genomic DNA as a template. The promoter of the β-lactamase gene was amplified using the primers AP-F/R (with *Hind* III at the 5′-end and *Xba* I at the 3′-end) (Table S1) and the pMD18-T vector as a template, and then the above two gene fragments were fused by T4 DNA ligase after *Hind* III digestion. After digestion by *Xba* I, the fused fragment was cloned into the pMD18-T vector to obtain recombinant pMD18-Mrc. After verification by DNA sequencing, pMD18-Mrc was transformed into *E. coli* DH5α (TSINGKE, China) for heterogeneous expression of FKQ53_RS21695, and the recombinant strain was named Mrc-3.

### Real-time quantitative PCR analysis

Total RNA isolation and RT-qPCR were performed as described previously (Fu et al. 2017). *P. pnomenusa* M202, *E. coli* M202-TC1, and *E. coli* Mrc-3 were harvested from broth medium at 24 h in the presence of 16 mg/L polymyxin B, and then rapidly frozen in liquid nitrogen. Total RNA was extracted using an RNA extraction kit (SBSBIO, Beijing, China), and then treated with Turbo DNA-free reagents (ABI Ambion, Austin, TX, USA). RT-qPCR assays were performed on a Roche Light Cycler 480 instrument using SYBR Green Mix (Toyobo, Osaka, Japan). Relative quantities of cDNA were normalized to the amounts of the 16S rRNA genes. The primers used are listed in Table S1.

### Determination of the MICs with an efflux pump inhibitor

The MICs of polymyxin B for the Mrc-3 strain were calculated by the broth microdilution procedure as described above, and then the MIC of polymyxin B in the presence of efflux pump inhibitors (EPIs) was also calculated. Verapamil, carbonyl cyanide (m-chlorophenyl) hydrazone (CCCP), dihydrochloride (PAβN), and reserpine (RES) were used as EPIs at final concentrations of 8.0 mg/L, 0.1 mg/L, 8.0 mg/L, and 8.0 mg/L, respectively.

### Molecular docking of FKQ53_RS21695 with polymyxin B

Transmembrane (TM) regions were identified by TMHMM 2.0 (https://services.healthtech.dtu.dk/service.php? TMHMM-2.0). Protein homology modeling and molecular docking were performed as previously described (Zong et al. 2020). The homology model constructed for the selected MFS transporter was analyzed using Rosetta software (Leman et al. 2020) and Discovery Studio (Biovia 2017). Hydrophobic surface features were analyzed by Discovery Studio (Biovia 2017). The structure of polymyxin B, as a ligand, was obtained from Chemspider (http://www.chemspider.com/). The binding of FKQ53_RS21695 with polymyxin B was modeled using the CDOCKER protocol of Discovery Studio 2.0 (Biovia 2017). The chemical bonds between FKQ53_RS21695 and polymyxin B were demonstrated by 3D and 2D methods.

## Results

### Polymyxin B resistance of the multidrug-resistant *P. pnomenusa* M202

*P. pnomenusa* M202 was isolated from hospital sewage under 8 mg/L polymyxin B selection pressure. MIC analysis of nine antibiotics, including a series of β-lactam, fluoroquinolone, tetracycline, chloramphenicol, aminoglycoside, sulfonamide, carbapenem, and glycopeptide antibiotics, was performed, and the results revealed that M202 was an MDR strain (Table S2). Interestingly, M202 showed high resistance to polymyxin B (MIC: 96 mg/L), which is the last-line therapeutic option against MDR bacteria associated with COVID-19 coinfections or other serious infections.

**Fig. 1:**
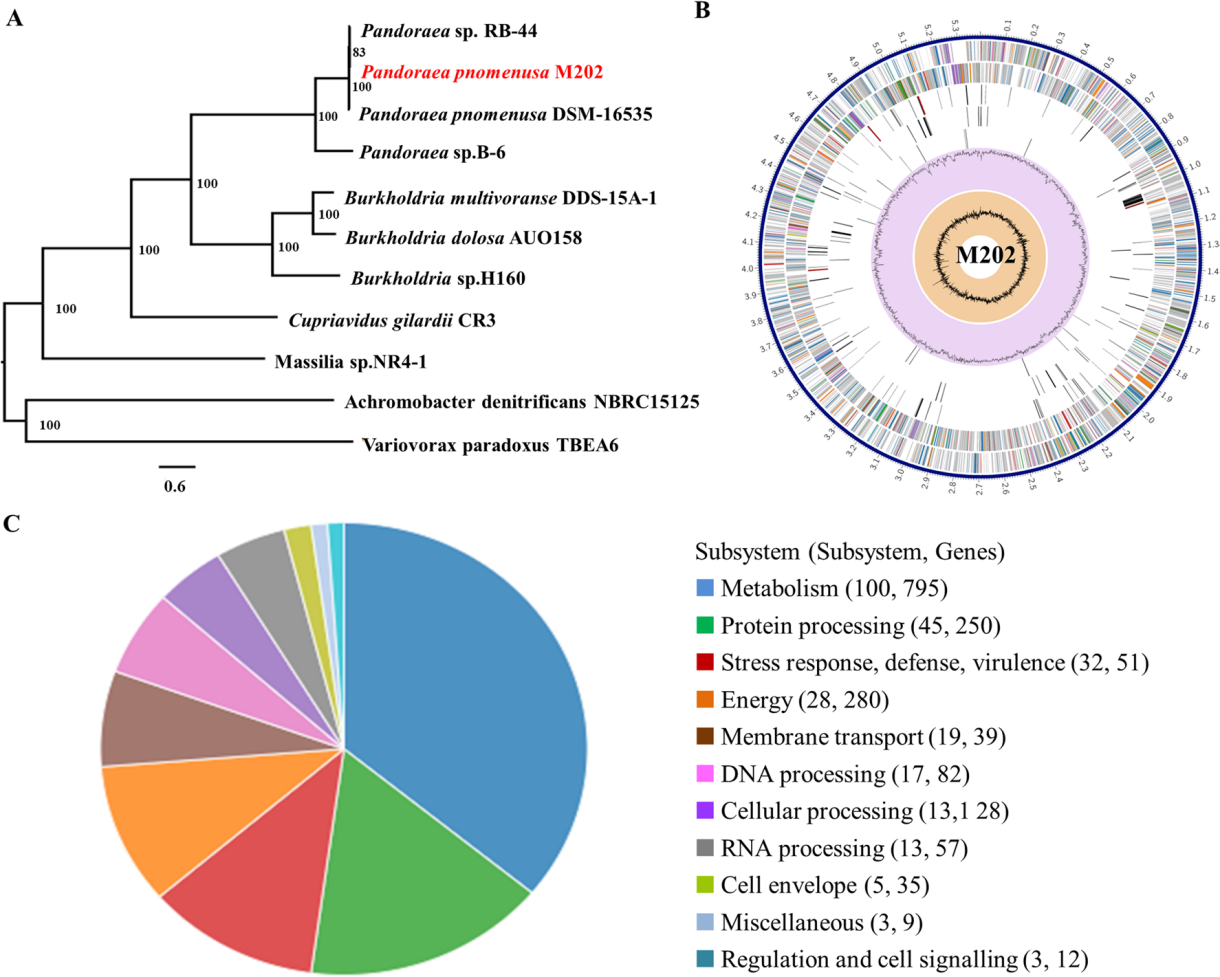
Identification and genomic characteristics of M202. **A** Neighbor-joining tree generated on the basis of 16S rDNA gene sequences of M202. **B** PATRIC annotation of the genome of M202. **C** Subsystem analysis of the M202 genome

Isolated strain M202 was identified as *P. pnomenusa* (Fig. 1A), which is an opportunistic pathogen. M202 harbors a 5.39 Mb circular chromosome with 64.79% GC content (Fig. 1B). The complete genome sequence was annotated, and 1165 functional proteins were assigned Enzyme Commission (EC) numbers, 1013 were assigned Gene Ontology (GO) terms, and 910 were mapped to KEGG pathways (Table S3). Subsystem analysis of the PATRIC annotations indicated that M202 contained 51 stress response, defense, and virulence genes and 39 membrane transport genes (Fig. 1C). The presence of twenty-one virulence factors (Table S4) and two type III secretion systems (Table S5) indicated the risk of clinical infection. Three prophage regions, region 1 (incomplete), region 2 (intact), and region 3 (incomplete), were identified (Table S6). These genes involved in the phage lytic cycle (including holin and tail proteins, gp6-like head-tail connector protein) may increase the possibility resistance transfer (Hernández-Mendoza et al. 2022). Additionally, abundant mobile genetic elements (MGEs), including thirty-nine insertion sequences (ISs) with transposases and five integrases (Table S7), showed that the diversity of the M202 genome may be attributed to horizontal gene transfer and that M202 also has the ability to spread antibiotic resistance.

### Antimicrobial resistance genes and potential antibiotic genomic islands in M202

Genes that are responsible for resistance to β-lactam, carbapenem, and tetracycline, including β-lactamase, AmpC, OXA-62, MBL metallo-hydrolase, and ribosome protection-type encoding genes, could be identified (Table 1). In addition, a considerable number of efflux pump genes were identified, and the major facilitator superfamily (MFS) and ATP-binding cassette (ABC) transporters were prominent classes, with 128 and 277 related genes, respectively (Table 1). In addition, lipopolysaccharide modification gene *pmrK*, an UDP phosphate-alpha-4-amino-4-deoxy-L-arabinose arabinosyl transferase encoding gene which was reported to be the main mechanism of polymyxin resistance in gram-negative bacteria (Moffatt et al. 2019), was also found in the genome of M202 (Table 1).

**Table 1:** Antibiotic resistance- and gene transfer-related genes in the M202 genome

Category	Class/subgroup	Protein	Number of related genes
β-Lactamase	Class B	MBL	10
	Class C	AmpC	1
	Class D	OXA-62	1
Tetracycline resistance	Ribosome protection type	Tcr	2
Polymyxin resistance	Lipopolysaccharide modification	*pmrK*-like	1
Efflux pumps	MFS	Transporter	128
	RND	Transporter	13
	SMR	Transporter	2
	MATE	Transporter	4
	ABC	Transporter	277
Permeability defects	Porin	Outer membrane protein	19
IS	IS256	Transposase	6
	IS3	Transposase	22
	IS30	Transposase	5
	DDE-type	Integrase/transposase/recombinase	1
	Ungrouped	Transposase	4
Integrase	Tyrosine-type	Integrase	3
	Site-specific	Integrase	2
Recombinase	Ungrouped	Recombinase	1
	RecA	Recombinase	1
	XerC	Recombinase	1
DNA translocase	FtsK	Translocase	2

*MFS*, major facilitator superfamily; *RND*, resistance nodulation division; *SMR*, small multidrug resistance; *MATE*, multidrug and toxic compound extrusion; *ABC*, ATP-binding cassette transporter

**Fig. 2:**
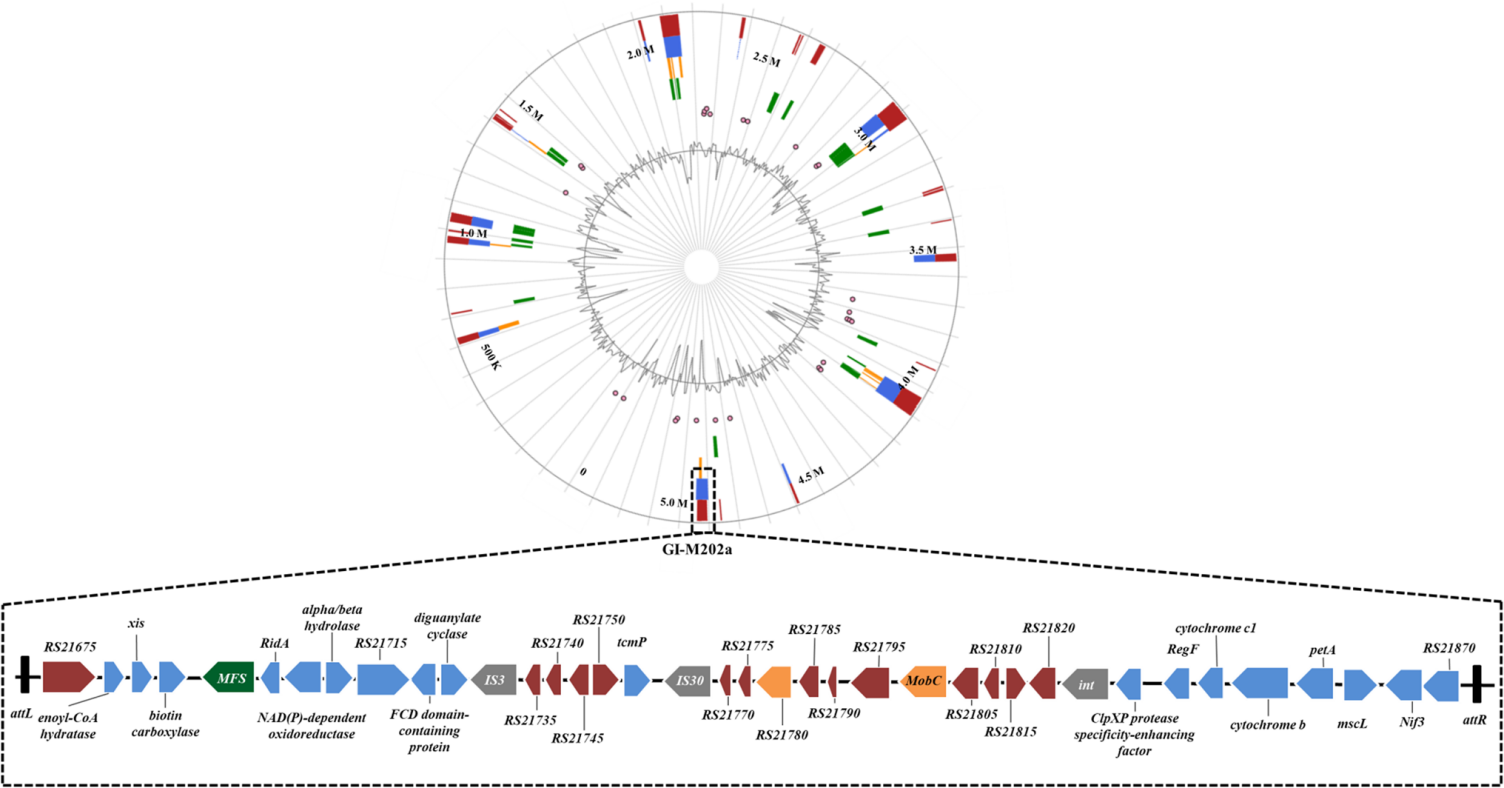
Identification of GI-M202a in the M202 genome. Top image, GIs predicted by IslandViewer 4. Putative GIs predicted by the SIGI-HMM method (yellow squares) or IslandPath-DIMOB method (blue squares). The integrated results are indicated by red squares. Bottom line, gene arrangement in GI-M202a. Genes are denoted by arrows. MFS transporter, green arrows; DNA transfer-related genes, gray arrows; genes of unknown function, purple arrows; type IV secretion system, orange arrows; others, blue arrows

To analyze the transfer and polymyxin resistance mechanism of M202, mobilizable GIs were investigated. Considered that *pmrK* (from 3,208,569 to 3,210,299 bp in genome) was not located in any GI, it is not the reason for polymyxin B resistance transfer ability. Thus, potential mobilizable GIs harboring antibiotic resistance genes were analyzed. Two overlapping regions, the first ranging from nucleotide positions 4904719 to 4940759 and the second from positions 4924191 to 4934831, were annotated as potential antibiotic GIs by IslandPath-DIMOB and SIGI-HMM, respectively. Further alignment analysis indicated that a single GI most likely extended from position 4904719 to 4942265, and this region was named GI-M202a (Fig. 2). Sequence examination further suggested that integration of GI-M202a may have occurred within the intergenic sequence between FKQ53_RS21670 and FKQ53_RS21875 and that this resulted in the formation of the incomplete attachment (att) sites GCTTTTATA and GCTTTTTTAT, which are similar to attL and attR of phage region 1 (Rutherford et al. 2013). Thirty-seven ORFs were identified in GI-M202a (Fig. 2); among them, four horizontal gene transfer elements, including two transposases (one of the IS3 family and the other of the IS30 family), two type IV conjugative transfer proteins (including a shufflon-specific DNA recombinase), one mobilization relaxosome protein (MobC), and one large-conductance mechanosensitive channel (FKQ53_RS21860), were predicted. In addition, one MFS transporter (FKQ53_RS21695) that might be related to antibiotic resistance was identified.

### An MFS transporter (FKQ53_RS21695) is responsible for polymyxin B resistance

**Fig. 3:**
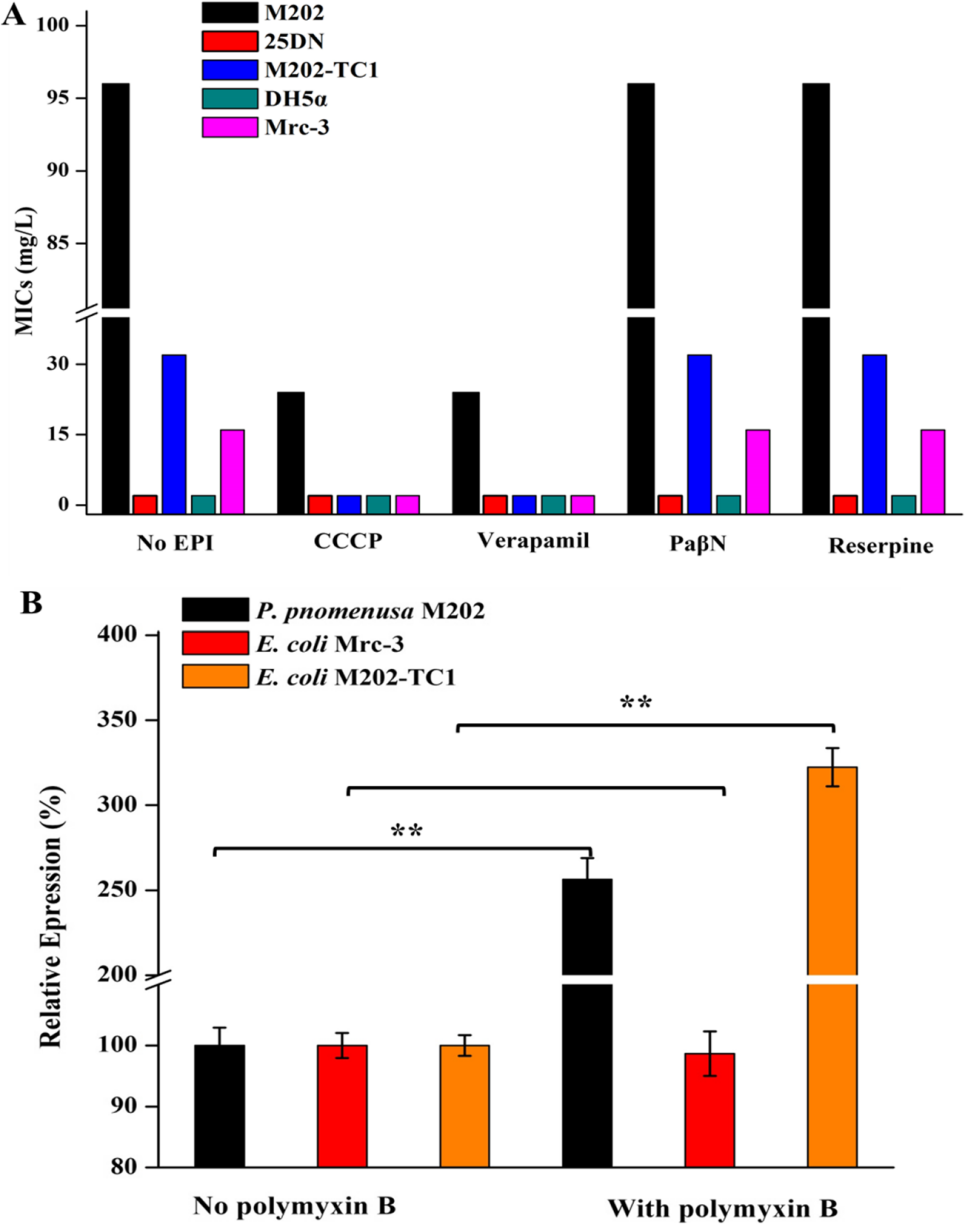
**A** MICs of polymyxin B for different strains. M202-TC1: transconjugant of M202 and *E. coli* 25DN; Mrc-3: DH5α with heterogeneous expression of FKQ53_RS21695. **B** RT-qPCR analyses of FKQ53_RS21695 gene in *P. pnomenusa* M202, *E. coli* M202-TC1, and *E. coli* Mrc-3. **: *p* < 0.001

PCR and DNA sequencing analysis confirmed that GI-M202a had been transferred to M202-TC1 (Fig. S1), indicating that the transferred gene conferring resistance to polymyxin B was in GI-M202a and was responsible for the polymyxin B resistance of M202-TC1. Considering that GI-M202a harbored one MFS transporter (FKQ53_RS21695), the EPIs CCCP and verapamil were used to evaluate efflux-mediated polymyxin B resistance as described previously (Fu et al. 2020). The MICs of polymyxin B for *P. pnomenusa* M202 and the *E. coli* transconjugant M202-TC1 clearly decreased from 96 to 24 mg/L and 24 mg/L in the presence of CCCP and verapamil, while PaβN and reserpine did not affect the MICs (Fig. 3A), suggesting that the MFS transporter (FKQ53_RS21695) in GI-M202a might be associated with polymyxin B resistance transfer.

To determine whether the MFS transporter (FKQ53_RS21695) is responsible for the polymyxin B resistance of M202, the encoding gene FKQ53_RS21695 was heterogeneously expressed in *E. coli* DH5α, and the recombinant strain was named Mrc-3. MIC analysis showed that Mrc-3 acquired resistance to polymyxin B (MIC 16 mg/L), and CCCP or verapamil clearly reduced the MICs of polymyxin B for the Mrc-3 strain to 2.0 mg/L, respectively (Fig. 3A). Real-time quantitative PCR analyses showed that expressions of FKQ53_RS21695 were higher in the presence of polymyxin B than the control group, both in *P. pnomenusa* M202 (increased by 2.56-fold) and *E. coli* M202-TC1 (increased 3.22-fold) (Fig. 3B). Moreover, expressions of FKQ53_RS21695 in *E. coli* Mrc-3 were detected, although it showed no significant difference between with and without polymyxin B. This result indicating that the MFS transporter FKQ53_RS21695 contributes polymyxin B resistance and transfer.

### Overall structure of the MFS transporter FKQ53_RS21695

**Fig. 4:**
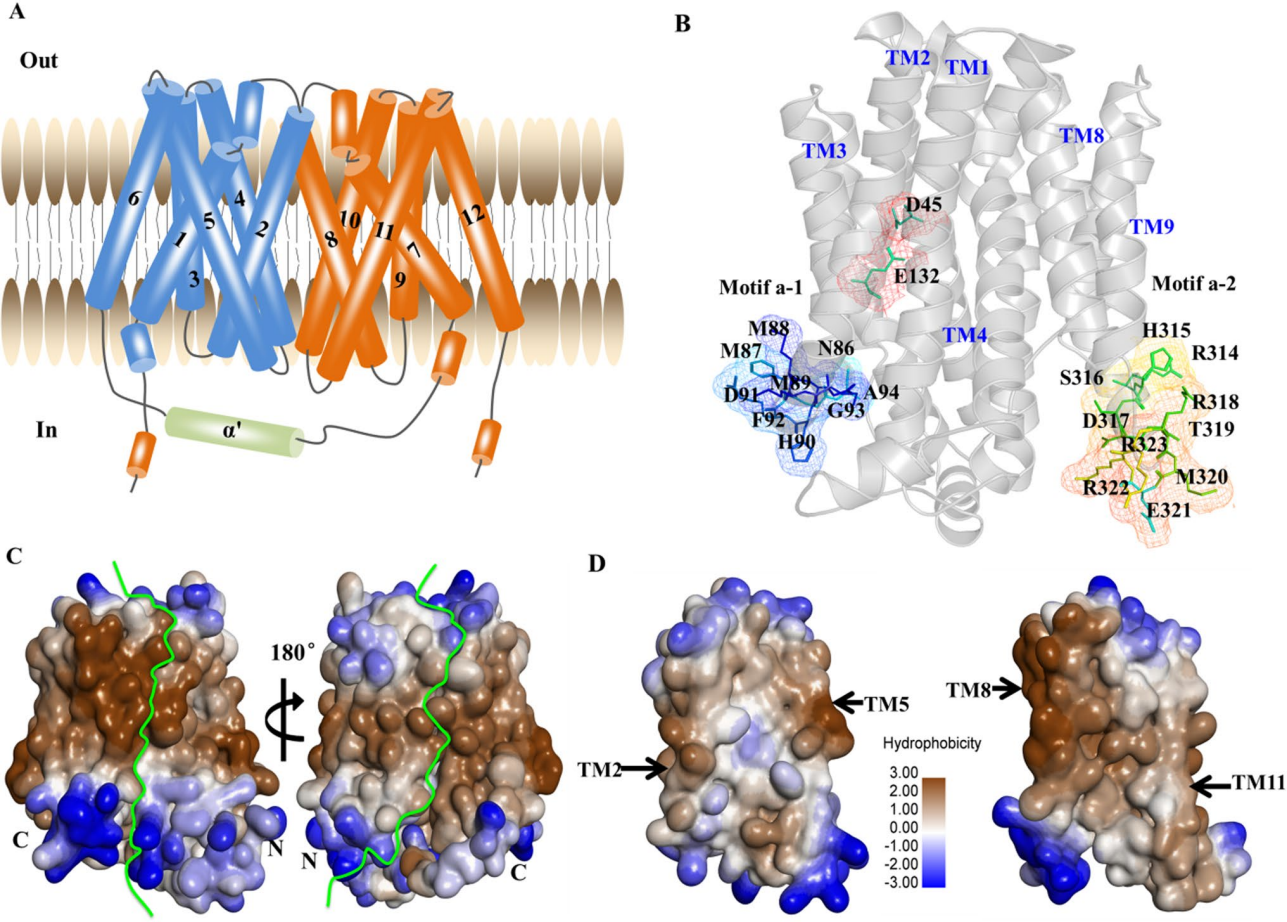
Overall structure of the MFS transporter FKQ53_RS21695 of GI-M202a. **A** Cartoon representation of the FKQ53_RS21695 structure. The N-domain, C-domain, and α′ are represented in blue, orange, and green, respectively. Numbers indicate the TM helixes. **B** Active conformation of motif A and protonation/deprotonation-related residues. **C** The overall hydrophobic surface of FKQ53_RS21695. **D** The cavity-facing sides of the N and C domains have contrasting surface electrostatic potentials. The figure was generated using DS

The MFS family protein FKQ53_RS21695 in GI-M202a consists of 450 amino acid residues. Similar to previously reported MFS transporter structures (Li et al. 2015), homologous modeling showed that the crystal structure of FKQ53_RS21695 contains 12 TM helices (TMs 1–12) and forms two domains (C domain and N domain) (Fig. 4A). The two domains are connected by a 34-residue linker (residues 223–246) containing a three-turn amphipathic α-helix as well as an extended loop.

Most MFS transporters contain the signature motif A, “G^(+1)^xlaD^(+5)^ rxGR^(+9)^kp” (Paulsen et al. 1996; Jiang et al. 2013). However, motif A-1 of FKQ53_RS21695, located in the loop connecting TMs 2 and 3 (L2–3, residues 86–95), was identified as “NMMMHRFGAR”. Similarly, motif A-2 was “RHSDRTMERR” and was located in the loop connecting TMs 8 and 9 (L8–9, residues 314–323) (Fig. 4B). This unusual motif made TM2 distant from TM11 and unable to form a close helix-helix contact, as also observed for *E. coli* YajR (Jiang et al. 2013). However, Arg^95^, which was proven to be important to the thermal stability and transport activity of MFS efflux proteins in YajR (Arg77) (Jiang et al. 2013) and TetA (Arg71) (Bannam et al. 2004), was conserved. Unlike the enriched Asp or Glu to be deprotonated during transport (Dang et al. 2010; Jiang et al. 2013; Heng et al. 2015; Leano et al. 2019), FKQ53_RS21695 maintained Asp^45^ and Glu^132^, which are located on the third helical turn of the corresponding TM1 and TM4 in the two internal structural repeats of the N domain amphipathic cavity (Fig. 4B). Therefore, only these residues can undergo cycles of protonation and deprotonation along the transport path.

The N and C domains of FKQ53_RS21695 have hydrophobic surface features. The N domain is capped by positively charged residues on the periplasmic side and by negatively charged residues on the cytoplasmic side (Fig. 4C). In addition, FKQ53_RS21695 has a central cavity in the TM core between the N and C domains. The N domain with a slight hydrophilic surface and C domain with a hydrophilic surface give rise to the amphipathic cavity of the TM core (Fig. 4D).

### Mechanism of polymyxin B efflux mediated by the MFS transporter FKQ53_RS21695

**Fig. 5:**
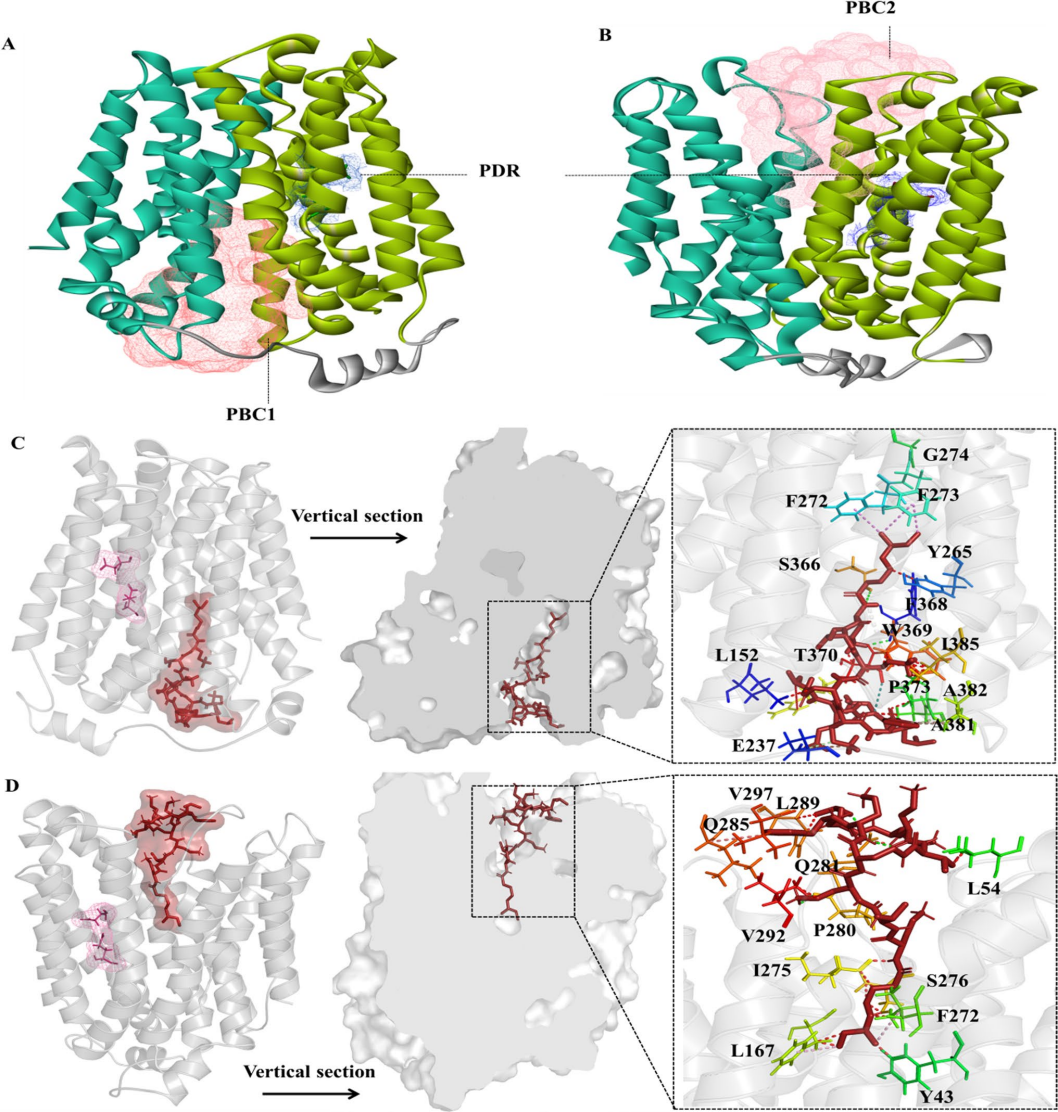
Polymyxin B efflux mediated by the MFS transporter FKQ53_RS21695. **A** PBC in the inward-open conformation. **B** PBC in outward-open conformation. PBC, polymyxin B binding cavity; PDR, protonation and deprotonation residues. **C** Interactions of PBC with polymyxin B in the inward-open conformation. **D** Interactions of PBC with polymyxin B in the outward-open conformation. Left: location of polymyxin B; right: interacting amino acid residues and chemical bonds

To analyze the details of polymyxin B efflux mediated by the MFS transporter FKQ53_RS21695, the interactions between polymyxin B and FKQ53_RS21695 were analyzed by molecular simulation. The outward-open and inward-open conformations of FKQ53_RS21695 showed the polymyxin B binding cavities (Fig. 5). In the inward-open conformation, the polymyxin B binding cavity (PBC1) was located in the central cavity in the TM core between the N and C domains. PBC presented a subulate form, which was embedded in the central cavity between the N and C domains (Fig. 5A). Similar to the inward-open conformation, PBC2 also presented subulate forms in the outward-open conformation (Fig. 5B). The cuspidal termini of PBC, in both outward-open and inward-open conformations, were located close to the protonation and deprotonation residues (PDR) (Fig. 5A–B). These results indicated that polymyxin B may “turn around” in the amphipathic cavity of the TM core during the conformational transition of FKQ53_RS21695.

Polymyxin B is an amphipathic lipopeptide molecule that contains a hydrophobic fatty acyl group at the N-terminus and a hydrophilic heptapeptide (Cai et al. 2015). The fatty acyl group at the N-terminus and the side chains of the residues at positions 6 (p6) and 7 (p7) contribute to the apolar portion (Cai et al. 2015). During efflux, the fatty acyl group inserts into the amphipathic cavity of the TM core for inward-open conformation and partially into the hydrophobic region. Phe^273^, Tyr^43^, Phe^272^, and Tyr^265^ of FKQ53_RS21695 were the main contributors located in binding cavities for fatty acyl group binding by Pi-alkyl and unfavorable bump interactions (Fig. 5C). The peptide fragment of FKQ53_RS21695 was bound by residues of TM10 and TM11 mainly via unfavorable bump interactions (Fig. 5C). This nature of the interaction allowed the peptide fragment to easily escape the binding, leading to a change in the orientation. After protonation and conformational change from inward-open to outward-open, the binding of polymyxin B moved from Phe^273^, Tyr^43^, Phe^272^, and Tyr^265^ to the adjacent amino acid residues Phe^272^, Tyr^43^, Leu^167^, Ser^276^, and Ile^275^. Moreover, the unfavorable bump interactions changed to the main chemical bonds binding FKQ53_RS21695 and polymyxin B, both the fatty acyl group and peptide fragment (Fig. 5D). Thus, separation from the outward-open conformation of FKQ53_RS21695 became easier than that from the inward-open conformation.

**Fig. 6:**
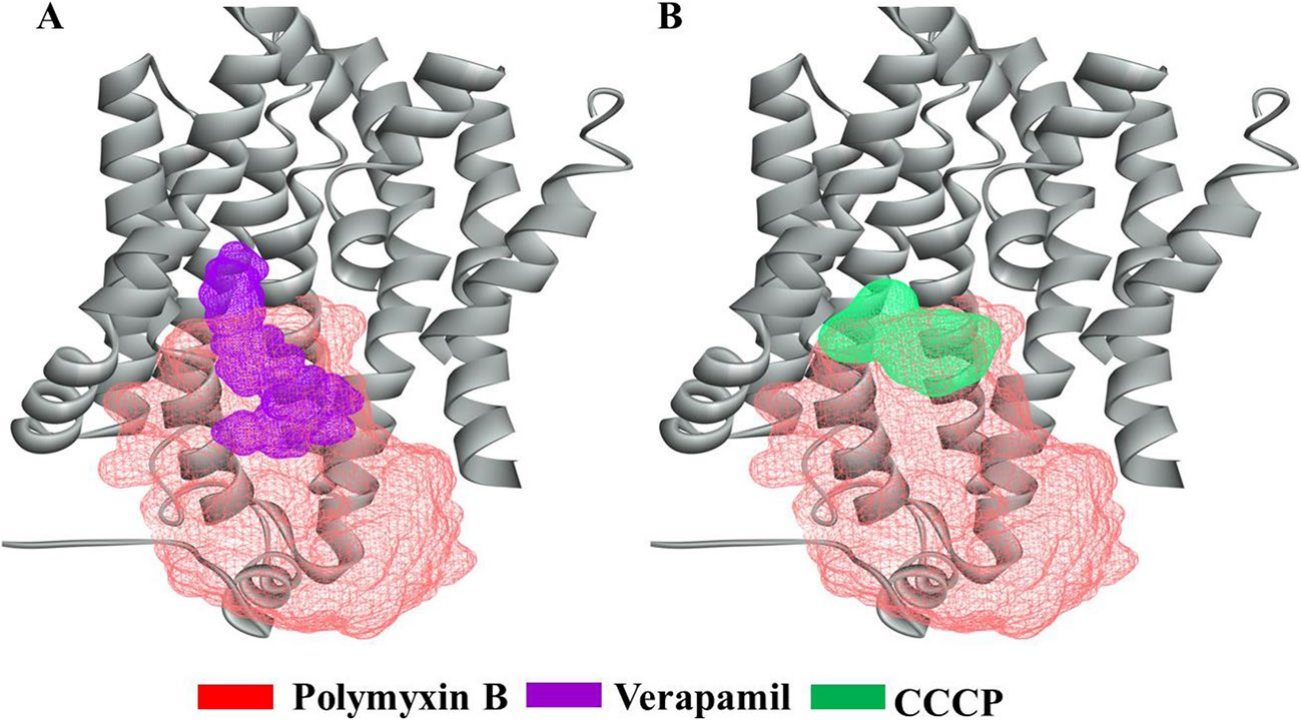
Binding cavities for verapamil and CCCP in FKQ53_RS21695. **A** Locations of the polymyxin B- and verapamil-binding cavities. **B** Locations of the polymyxin B- and CCCP-binding cavities

Moreover, FKQ53_RS21695 showed binding with verapamil and CCCP. Interestingly, these binding cavities for EPIs in FKQ53_RS21695 partly overlapped with the polymyxin B binding cavity (Fig. 6). This indicated that the inhibitory effect of verapamil and CCCP on polymyxin B resistance may be attributed to binding site competition. The key EPI-binding amino acids were Phe^136^, Arg^46^, Tyr^160^, and Phe^272/273^ (Fig. S2). The polymyxin B efflux-related key amino acids, Phe^272^ and Phe^273^, bind to verapamil and CCCP, leading to attenuation of polymyxin B binding and “turn around” movement in the amphipathic cavity of FKQ53_RS21695.

## Discussion

Gram-negative microorganisms are associated with coinfections in patients with COVID-19 (Povoa et al. 2020; Sehgal et al. 2021; Singh et al. 2021; Cubides-Diaz et al. 2022). *Pandoraea* species, which are gram-negative bacteria, can cause bacteremia and lung function decline (Lin et al. 2019). *P. pnomenusa* superinfection in a patient with COVID-19 pneumonia was found in 2022 in Colombia (Cubides-Diaz et al. 2022). *Pandoraea* spp. are resistant to many antibiotics, which makes the treatment of *Pandoraea*-related infections more complicated (Lin et al. 2019). Polymyxin B is thought to be a last-line therapeutic option against MDR gram-negative bacteria (Katagiri et al. 2022) and has been used to treat coinfections in patients with COVID-19. However, we isolated *P. pnomenusa* strain M202 with polymyxin B resistance from hospital sewage, which caught our attention. Hence, exploration of resistance to the emergency antibiotic polymyxin B, after 3 years of the pandemic, is urgently needed.

Moreover, *P. pnomenusa* M202 is also resistant to ampicillin, cefixime, tetracycline, amikacin, sulfamethoxazole, and meropenem. This multidrug-resistant strain contains several antibiotic resistance genes, such as β-lactamase encoding genes, Tcr (tetracycline-resistant gene), and efflux pump genes. Especially, carbapenem-hydrolyzing oxacillinase OXA-62, which was found to be a novel mechanism of carbapenem resistance in *P. pnomenusa* (Schneider et al. 2006), was also identified in M202 genome (Table 1). In addition, multidrug-resistant *P. pnomenusa* strains RB-44, which was reported at 2014 in Malaysia (Ee et al. 2014), also harbored plenty of antibiotic resistance genes, including class A, B, C, and D β-lactamase encoding genes, tetracycline-resistant genes, and efflux pumps genes (Table 1). These data indicate a potential reservoir of antibiotic resistance genes in this species and a further complication in the treatment of infections caused by *P. pnomenusa*.

Polymyxins have been used to treat MDR gram-negative bacterial infections. It is generally accepted that gram-negative selectivity is mediated by initial interactions with the outer membrane with negatively charged lipopolysaccharides (LPSs), where the cationic polypeptide portion of polymyxin electrostatically binds. LPSs are the predominant (or only) surface lipids of the outer membrane in gram-negative bacteria, and the binding is assisted by the interactions of the lipid tail with the fatty acids of the lipid A moiety of the LPS molecules (Fernandez et al. 2013; Trimble et al. 2016). The polymyxin resistance mechanism mainly involves alterations to reduce the net negative charge or fluidity of LPS (summarized in Trimble et al. 2016). However, despite the membrane being the sole target for polymyxins, alternative or additional mechanisms of action likely contribute to the antibacterial activity of polymyxins. Intracellular components such as the 16S A-site of *E. coli* ribosomes (McCoy et al. 2013) and the oxidative stress response gene *soxS* (Dong et al. 2015) are potential targets. However, polymyxin-resistant pathogens pose a serious threat to human health. In this study, the mobilizable antibiotic resistance island GI-M202a was found and identified as a novel antibiotic resistance island that confers polymyxin B resistance. This enhanced risk of polymyxin B resistance spreading to the environment needs to be monitored closely, especially in the context of COVID-19 coinfections, before a better therapeutic regimen is developed.

**Fig. 7:**
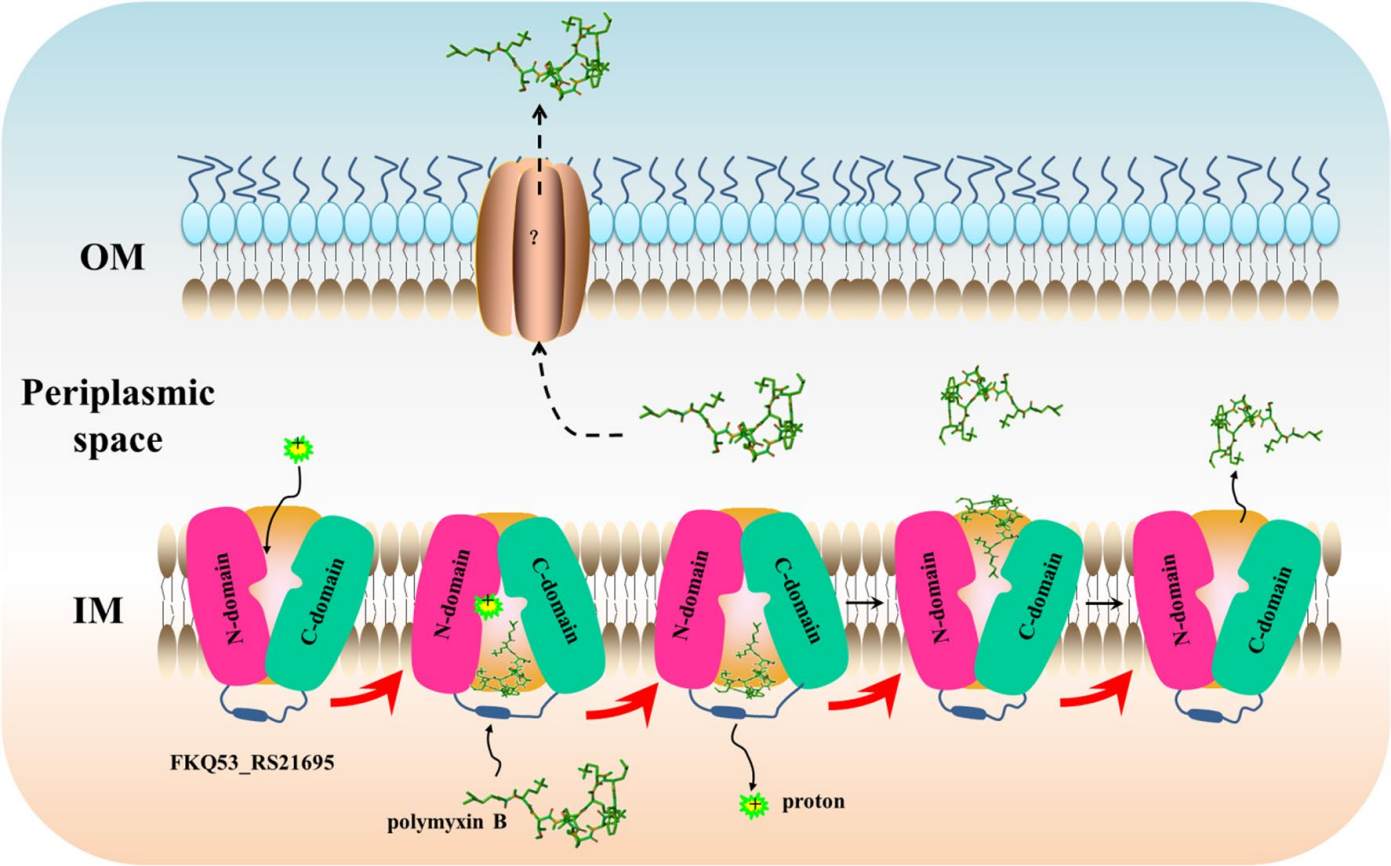
Proposed polymyxin B transport mechanism mediated by FKQ53_RS21695. Polymyxin B “turn around” transport mode

MFS transporters constitute the largest class of secondary transporters and share a similar folding topology and function (Huang et al. 2003; Jiang et al. 2013). MFS proteins are believed to switch between two major conformations, inward and outward, which differ by an ∼40° rotation of one domain relative to the other (Jiang et al. 2013). The conformational changes can be further regulated by substrate binding inside the cavity or at allosteric sites on either side of the TM core, depending on the direction of substrate transport (Guan and Kaback 2004; Jiang et al. 2013). Most MFS proteins are located in the IM and transport drugs from the cytosol to the periplasm (Li et al. 2015). Similar to the typical MFS structure, FKQ53_RS21695 contains a 12 TM helix core composed of two six-helix rigid domains forming a central TM channel and two antibiotic binding cavities (each present in each conformation) with different interactions with and affinities for antibiotics, similar to ABC transporters coupled with ATP hydrolysis (Orelle et al. 2003; Hofmann et al. 2019). The pathway by which polymyxin B is discharged from the periplasm to the extracellular space may need the assistance of a membrane fusion protein or porin, similar to the EmrAB-TolC system (Tanabe et al. 2009; Blair et al. 2014), resistance-nodulation-division transporter ArcAB-TolC system (Nolivos et al. 2019), or HlyBD-TolC system (Holland et al. 2016). In this study, we found that the MFS transporter FKQ53_RS21695 could bind and facilitate efflux of polymyxin B. During the efflux process, the polymyxin B fatty acyl group inserts into the hydrophobic region of the TM core with Pi-alkyl and unfavorable bump interactions. Then, polymyxin B turns over around Tyr^43^ to externally display the peptide group, accompanied by the generation of more unfavorable bumps in the outward conformation. Finally, the mechanism by which polymyxin B passes through the FKQ53_RS21695 TM core is proposed (Fig. 7). In addition, a previous study revealed that insertional inactivation of the MFS gene *kpnGH* resulted in increased susceptibility to polymyxin-B in *Klebsiella pneumoniae* (Srinivasan et al. 2014), also indicating the polymyxin B function of the MFS transporter. However, this antibiotic transport mode of antibiotic binding cavities associated with proton transport needs further investigation.

## Conclusions

The results of this study reflect that the MDR bacterial *P. pnomenusa* M202 is an effective disseminator of polymyxin B resistance. The MFS efflux pump FKQ53_RS21695 in GI-M202a is responsible for the polymyxin B resistance. FKQ53_RS21695 facilitates polymyxin B efflux through its amphipathic core, which is composed of TM regions. The penetrating power is related to different interactions between MFS transporters and polymyxin B when FKQ53_RS21695 presents inward and outward conformations.

## Supplementary information

ESM 1 (DOC 1004 kb)

## Data Availability

The datasets supporting the conclusions of this article are available from the lead authors upon reasonable request.

## Declarations

### Nucleotide sequence accession number

Complete sequences of the *Pandoraea pnomenusa* M202 genome were deposited in GenBank under accession no. CP041237.
