# Supplementary material for: An efflux pump in genomic island GI-M202a mediates the transfer of polymyxin B resistance in Pandoraea pnomenusa M202
Source: Int Microbiol. 2023 Jun 15;27(1):277–90. doi: 10.1007/s10123-023-00384-8 (PMC10266961; doi:10.1007/s10123-023-00384-8)
Supplement: Supplementary file 1 — (DOC 1004 kb) [file 10123_2023_384_MOESM1_ESM.doc]

**Table S1.** Primers sequence (5′→3′) used in this study

| Primer ID | | | Primer sequences (5’-3’) |
| --- | --- | --- | --- |
| **For mating experiments verification** | | | |
| V1-F | ATGCCTGCCCCCTATGTCG | | |
| V1-R | CTAGCCTGAAATCCGATCCGTC | | |
| V2-F | CAGTGCGCATAGGCGCTG | | |
| V2-R | ATGAAGCCATCCGAGTCAAGTG | | |
| V3-F | TCAGATGGGGGAATTCGCTG | | |
| V3-R | ATGGCACAGGTCATTCCCGT | | |
| **For FKQ53_RS21695 recombination** | | | |
| MRC-F | | AATATTGAAAAAGGAAGAGTATGAAGCCATCCGAGTCAAGTG | |
| MRC-R | | aagcttCAGCGCCTATGCGCACTGA | |
| AP-F | | aagcttTCTAGAGCCTCGTGATACGCCTATTT | |
| AP-R | | ACTCTTCCTTTTTCAATATT | |
| **For FKQ53_RS21695 real-time PCR** | | | |
| RT-F | | aaggatgaacggtatggcgct | |
| RT-R | | ccatcaaccagaccatcggc | |

**Table S2.** MICs of antibiotics for *P. pnomenusa* M202 (mg/L)

| Ampicillin | Cefixime | Tetracycline | Ciprofloxacin | Florfenicol | Amikacin | Polymyxin B | Sulfamethoxazole | Meropenem |
| --- | --- | --- | --- | --- | --- | --- | --- | --- |
| >128^R^ | >128^R^ | >128^R^ | 8^R^ | 8^S^ | >128^R^ | 96^R^ | >128^R^ | 16^R^ |

Abbreviations: R: Resistant; S: Susceptible

**Table S3.** General features of the M202 genome

| Features | Genome |
| --- | --- |
| Total number of base pairs | 5,389,809 bp |
| G+C content (%) | 64.79 |
| Total genes | 4,959 |
| Repeat Regions | 65 |
| tRNA | 64 |
| rRNA | 12 |
| Hypothetical proteins | 1,228 |
| Proteins with functional assignments | 3,731 |
| Proteins with EC number assignments | 1,165 |
| Proteins with GO assignments | 1,013 |
| Proteins with KEGG assignments | 910 |
| Proteins with PATRIC genus-specific family (PLfam) assignments | 4,687 |
| Proteins with PATRIC cross-genus family (PGfam) assignments | 4,763 |

Abbreviations: EC, Enzyme Commission; GO, Gene Ontology database; KEGG, Kyoto Encyclopedia of Genes and Genomes.

**Table S4.** Predicted virulence factors of the M202

|  | Gene/locus tag | Coordinates (+/-) | Hit (Ha-value) | Product |
| --- | --- | --- | --- | --- |
| 1 | FKQ53_RS00740 | 173862..174125 (+) | [VFG000550](http://www.mgc.ac.cn/cgi-bin/VFs/gene.cgi?GeneID=VFG000550) (0.651) | (gb|NP_461810) (spaQ) type III secretion system minor export apparatus protein SpaQ [TTSS(SPI-1 encode) (VF0116)] |
| 2 | FKQ53_RS10155 | 2260542..2261444 (-) | [glxR](http://www.ncbi.nlm.nih.gov/protein/?term=glxR) (0.651) | Tartronic semialdehyde reductase |
| 3 | FKQ53_RS10165 | 2262673..2264439 (-) | [gcl](http://www.ncbi.nlm.nih.gov/protein/?term=gcl) (0.752) | Glyoxylate carboligase |
| 4 | FKQ53_RS10345 | 2300266..2301588 (+) | [VFG001381](http://www.mgc.ac.cn/cgi-bin/VFs/gene.cgi?GeneID=VFG001381) (0.682) | (gb|YP_177728) (icl) Isocitrate lyase Icl (isocitrase) (isocitratase) |
| 5 | FKQ53_RS17260 | 3917172..3918818 (-) | [VFG001855](http://www.mgc.ac.cn/cgi-bin/VFs/gene.cgi?GeneID=VFG001855) (0.713) | (gb|YP_094724) (htpB) Hsp60, 60K heat shock protein HtpB |
| 6 | FKQ53_RS22880 | 5160499..5161593 (-) | [VFG000036](http://www.mgc.ac.cn/cgi-bin/VFs/gene.cgi?GeneID=VFG000036) (0.713) | (gb|NP_878993) (bplC) lipopolysaccharide biosynthesis protein |
| 7 | FKQ53_RS22885 | 5161596..5162180 (-) | [VFG000037](http://www.mgc.ac.cn/cgi-bin/VFs/gene.cgi?GeneID=VFG000037) (0.707) | (gb|NP_878994) (bplB) probable acetyltransferase |
| 8 | FKQ53_RS22920 | 5169586..5170359 (-) | [VFG002377](http://www.mgc.ac.cn/cgi-bin/VFs/gene.cgi?GeneID=VFG002377) (0.736) | (gb|YP_001007269) (ddhA) glucose-1-phosphate cytidylyltransferase [O-antigen (VF0392)] |
| 9 | FKQ53_RS23270 | 5250775..5251773 (+) | [VFG002501](http://www.mgc.ac.cn/cgi-bin/VFs/gene.cgi?GeneID=VFG002501) (0.755) | (gb|YP_106857) (fliG) flagellar motor switch protein G |
| 10 | FKQ53_RS23280 | 5252761..5254179 (+) | [VFG002499](http://www.mgc.ac.cn/cgi-bin/VFs/gene.cgi?GeneID=VFG002499) (0.669) | (gb|YP_106855) (fliI) flagellum-specific ATP synthase FliI |
| 11 | FKQ53_RS23300 | 5256790..5257788 (+) | [VFG002491](http://www.mgc.ac.cn/cgi-bin/VFs/gene.cgi?GeneID=VFG002491) (0.729) | (gb|YP_106652) (fliM) flagellar motor switch protein FliM |
| 12 | FKQ53_RS23315 | 5258905..5259609 (+) | [VFG002494](http://www.mgc.ac.cn/cgi-bin/VFs/gene.cgi?GeneID=VFG002494) (0.676) | (gb|YP_106655) (fliP) flagellar biosynthesis protein FliP |
| 13 | FKQ53_RS23320 | 5259626..5259895 (+) | [VFG002339](http://www.mgc.ac.cn/cgi-bin/VFs/gene.cgi?GeneID=VFG002339) (0.753) | (gb|YP_001006752) (fliQ) flagellar biosynthetic protein FliQ |
| 14 | FKQ53_RS23355 | 5267051..5267833 (-) | [VFG002513](http://www.mgc.ac.cn/cgi-bin/VFs/gene.cgi?GeneID=VFG002513) (0.767) | (gb|YP_106902) (flgG) flagellar basal body rod protein FlgG |
| 15 | FKQ53_RS23375 | 5270734..5271138 (-) | [VFG002509](http://www.mgc.ac.cn/cgi-bin/VFs/gene.cgi?GeneID=VFG002509) (0.745) | (gb|YP_106898) (flgC) flagellar basal body rod protein FlgC |
| 16 | FKQ53_RS23410 | 5274759..5275487 (-) | [VFG002519](http://www.mgc.ac.cn/cgi-bin/VFs/gene.cgi?GeneID=VFG002519) (0.700) | (gb|YP_109887) (fliA) flagellar biosynthesis sigma factor |
| 17 | FKQ53_RS23425 | 5277637..5279742 (-) | [VFG002522](http://www.mgc.ac.cn/cgi-bin/VFs/gene.cgi?GeneID=VFG002522) (0.753) | (gb|YP_109890) (flhA) flagellar biosynthesis protein FlhA |
| 18 | FKQ53_RS23440 | 5281778..5282176 (-) | [VFG002525](http://www.mgc.ac.cn/cgi-bin/VFs/gene.cgi?GeneID=VFG002525) (0.746) | (gb|YP_109896) (cheY) chemotaxis protein CheY |
| 19 | FKQ53_RS23445 | 5282314..5283393 (-) | [VFG002526](http://www.mgc.ac.cn/cgi-bin/VFs/gene.cgi?GeneID=VFG002526) (0.777) | (gb|YP_109897) (cheB) chemotaxis-specific methylesterase |
| 20 | FKQ53_RS23475 | 5291205..5291720 (-) | [VFG002530](http://www.mgc.ac.cn/cgi-bin/VFs/gene.cgi?GeneID=VFG002530) (0.771) | (gb|YP_109901) (cheW) chemotaxis protein CheW |
| 21 | FKQ53_RS23495 | 5295457..5296317 (-) | [VFG002534](http://www.mgc.ac.cn/cgi-bin/VFs/gene.cgi?GeneID=VFG002534) (0.724) | (gb|YP_109905) (motA) flagellar motor protein MotA |

**Table S5.** Identified type III secretion apparatus of the M202 genome

|  | Start | Stop | Strand | Locus tag | Length | Protein name |  |  |
| --- | --- | --- | --- | --- | --- | --- | --- | --- |
| Region 1 | 908305 | 908523 | + | FKQ53_RS04115 | 72 | EscF/YscF/HrpA family type III secretion system needle major subunit |  |  |
|  | 908520 | 908942 | + | FKQ53_RS04120 | 140 | DUF1039 domain-containing protein | |  |
|  | 909337 | 910146 | + | FKQ53_RS04130 | 269 | EscJ/YscJ/HrcJ family type III secretion inner membrane ring protein | |  |
|  | 910032 | 910805 | + | FKQ53_RS04135 | 257 | hypothetical protein |  |  |
|  | 910781 | 911470 | + | FKQ53_RS04140 | 229 | hypothetical protein |  |  |
|  | 911463 | 912596 | + | FKQ53_RS04145 | 377 | MULTISPECIES: TyeA family type III secretion system gatekeeper subunit | |  |
|  | 912734 | 913291 | - | FKQ53_RS04150 | 185 | MULTISPECIES: hypothetical protein |  |  |
|  | 913365 | 913607 | - | FKQ53_RS04155 | 80 | MULTISPECIES: EscE/YscE/SsaE family type III secretion system needle protein co-chaperone | |  |
|  | 913579 | 915000 | - | FKQ53_RS04160 | 473 | EscD/YscD/HrpQ family type III secretion system inner membrane ring protein | |  |
|  | 915003 | 916634 | - | FKQ53_RS04165 | 543 | EscC/YscC/HrcC family type III secretion system outer membrane ring protein | |  |
|  | 916637 | 917134 | - | FKQ53_RS04170 | 165 | hypothetical protein |  |  |
|  | 917461 | 918120 | + | FKQ53_RS04175 | 219 | MULTISPECIES: two component system response regulator | |  |
|  | 918358 | 918741 | + | FKQ53_RS04180 | 127 | hypothetical protein |  |  |
|  | 918756 | 920831 | + | FKQ53_RS04185 | 691 | EscV/YscV/HrcV family type III secretion system export apparatus protein | |  |
|  | 920833 | 922197 | + | FKQ53_RS04190 | 454 | FliI/YscN family ATPase |  |  |
|  | 922173 | 922607 | + | FKQ53_RS04195 | 144 | hypothetical protein |  |  |
|  | 922669 | 923151 | + | FKQ53_RS04200 | 160 | hypothetical protein |  |  |
|  | 923141 | 924196 | + | FKQ53_RS04205 | 351 | hypothetical protein |  |  |
|  | 924253 | 924903 | + | FKQ53_RS04210 | 216 | EscR/YscR/HrcR family type III secretion system export apparatus protein | |  |
|  | 924923 | 925189 | + | FKQ53_RS04215 | 88 | MULTISPECIES: EscS/YscS/HrcS family type III secretion system export apparatus protein | |  |
|  | 925194 | 926036 | + | FKQ53_RS04220 | 280 | EscT/YscT/HrcT family type III secretion system export apparatus protein | |  |
|  | 926044 | 927267 | + | FKQ53_RS04225 | 407 | EscU/YscU/HrcU family type III secretion system export apparatus switch protein | |  |
| Region 2 | 158876 | 160138 | + | FKQ53_RS00660 | 420 | PrgH/EprH family type III secretion apparatus protein | |  |
|  | 160241 | 160510 | + | FKQ53_RS00665 | 89 | MULTISPECIES: EscF/YscF/HrpA family type III secretion system needle major subunit | |  |
|  | 160577 | 160864 | + | FKQ53_RS00670 | 95 | hypothetical protein | |  |
|  | 160861 | 161673 | + | FKQ53_RS00675 | 270 | MULTISPECIES: EscJ/YscJ/HrcJ family type III secretion inner membrane ring protein | |  |
|  | 161496 | 162287 | + | FKQ53_RS00680 | 263 | MULTISPECIES: type III secretion apparatus protein OrgA/MxiK | |  |
|  | 162244 | 163035 | + | FKQ53_RS00685 | 263 | hypothetical protein | |  |
|  | 163016 | 163345 | + | FKQ53_RS00690 | 109 | MULTISPECIES: hypothetical protein | |  |
|  | 163411 | 164196 | + | FKQ53_RS00695 | 261 | helix-turn-helix domain-containing protein | |  |
|  | 164193 | 166214 | + | FKQ53_RS00700 | 673 | EscC/YscC/HrcC family type III secretion system outer membrane ring protein | |  |
|  | 166072 | 167343 | + | FKQ53_RS00705 | 423 | YopN family type III secretion system gatekeeper subunit | |  |
|  | 167345 | 169432 | + | FKQ53_RS00710 | 695 | MULTISPECIES: EscV/YscV/HrcV family type III secretion system export apparatus protein | |  |
|  | 169461 | 170804 | + | FKQ53_RS00715 | 447 | MULTISPECIES: FliI/YscN family ATPase | |  |
|  | 170788 | 171249 | + | FKQ53_RS00720 | 153 | hypothetical protein | |  |
|  | 171231 | 172301 | + | FKQ53_RS00725 | 356 | hypothetical protein | |  |
|  | 172295 | 173215 | + | FKQ53_RS00730 | 306 | MULTISPECIES: YscQ/HrcQ family type III secretion apparatus protein | |  |
|  | 173205 | 173849 | + | FKQ53_RS00735 | 214 | MULTISPECIES: EscR/YscR/HrcR family type III secretion system export apparatus protein | |  |
|  | 173862 | 174125 | + | FKQ53_RS00740 | 87 | MULTISPECIES: EscS/YscS/HrcS family type III secretion system export apparatus protein | |  |
|  | 174127 | 174924 | + | FKQ53_RS00745 | 265 | SpaR/YscT/HrcT type III secretion system export apparatus protein | |  |
|  | 174926 | 176140 | + | FKQ53_RS00750 | 404 | EscU/YscU/HrcU family type III secretion system export apparatus switch protein | |  |
|  | 176959 | 177525 | + | FKQ53_RS00755 | 188 | MULTISPECIES: type III secretion system translocator chaperone SicA | |  |
|  | 177522 | 179243 | + | FKQ53_RS00760 | 573 | MULTISPECIES: hypothetical protein | |  |
|  | 179260 | 180300 | + | FKQ53_RS00765 | 346 | MULTISPECIES: hypothetical protein | |  |
|  | 180354 | 181544 | + | FKQ53_RS00770 | 396 | IpaD/SipD/SspD family type III secretion system needle tip protein | |  |

**Table S6.** Identified prophage regions of the M202 genome

|  | Region 1 | Region 2 | Region 3 |
| --- | --- | --- | --- |
| Length | 12.3Kb | 51.7Kb | 7.1Kb |
| Incomplete/ intact | Incomplete | Intact | Incomplete |
| Specific | Integrase, tail | terminase, portal, head, capsid, tail, virion, integrase, transposase | head, transposase |
| Position | 2088385-2100720 | 2088475-2140189 | 4918033-4925207 |
| Total protein number | 10 | 58 | 8 |
| Phage hit protein number | 6 | 41 | 6 |
| Hypothetical protein number | 4 | 15 | 1 |
| att sites | attL-1, attR-1  AAATAAAAACGT | attL-2, attR-2  CTGGATTGTGATTCCAGTCGTCGTGGGTTCGAGTCCCATCGGTCACCCCAAAAT | / |
| Phage species protein number | 6 | 26 | 4 |

/ : undetected

**Table S7.** Identified insertion sequences (IS) with transposases of M202

| Start | Stop | Strand | Locus tag | Length | Protein name |
| --- | --- | --- | --- | --- | --- |
| 6304 | 7554 | - | FKQ53_RS00025 | 416 | IS256 family transposase |
| 222904 | 223911 | + | FKQ53_RS00940 | 335 | MULTISPECIES: tyrosine recombinase XerC |
| 607800 | 608804 | + | FKQ53_RS02810 | 334 | tyrosine-type recombinase/integrase |
| 609370 | 610604 | + | FKQ53_RS02820 | 411 | IS3 family transposase |
| 963016 | 964554 | + | FKQ53_RS04415 | 512 | IS3 family transposase |
| 969016 | 970250 | - | FKQ53_RS04445 | 411 | IS3 family transposase |
| 1028615 | 1029852 | + | FKQ53_RS04640 | 412 | IS3 family transposase |
| 1037319 | 1038290 | - | FKQ53_RS04685 | 323 | IS30 family transposase |
| 1038393 | 1039579 | + | FKQ53_RS04690 | 395 | IS3 family transposase |
| 1040734 | 1041968 | - | FKQ53_RS04710 | 411 | IS3 family transposase |
| 1049033 | 1050049 | + | FKQ53_RS04745 | 338 | IS30 family transposase |
| 1050217 | 1051467 | - | FKQ53_RS04755 | 416 | IS256 family transposase |
| 1051406 | 1051774 | - | FKQ53_RS04760 | 122 | DDE-type integrase/transposase/recombinase |
| 1051820 | 1052928 | - | FKQ53_RS04765 | 369 | IS3 family transposase |
| 1054350 | 1055587 | + | FKQ53_RS04780 | 412 | IS3 family transposase |
| 1059432 | 1060448 | - | FKQ53_RS04805 | 338 | MULTISPECIES: IS30 family transposase |
| 1413862 | 1415024 | - | FKQ53_RS06285 | 387 | IS3 family transposase |
| 1554228 | 1554623 | - | FKQ53_RS06840 | 131 | MULTISPECIES: transposase |
| 1555350 | 1556165 | + | FKQ53_RS06845 | 271 | MULTISPECIES: IclR family transcriptional regulator |
| 1719158 | 1720408 | + | FKQ53_RS07575 | 416 | IS256 family transposase |
| 2135216 | 2136466 | - | FKQ53_RS09585 | 416 | IS256 family transposase |
| 2371094 | 2372211 | - | FKQ53_RS10635 | 372 | IS3 family transposase |
| 2377548 | 2378782 | - | FKQ53_RS10655 | 411 | IS3 family transposase |
| 2485829 | 2487013 | - | FKQ53_RS11085 | 394 | MULTISPECIES: site-specific integrase |
| 2972772 | 2973050 | - | FKQ53_RS13170 | 92 | transposase |
| 2974406 | 2975640 | + | FKQ53_RS13185 | 411 | IS3 family transposase |
| 3022763 | 3024000 | + | FKQ53_RS13380 | 412 | IS3 family transposase |
| 3024977 | 3026163 | - | FKQ53_RS13395 | 395 | IS3 family transposase |
| 3026553 | 3027569 | + | FKQ53_RS13400 | 338 | IS30 family transposase |
| 3028571 | 3029482 | + | FKQ53_RS13405 | 303 | site-specific integrase |
| 3540498 | 3541732 | - | FKQ53_RS15615 | 411 | IS3 family transposase |
| 3541838 | 3542946 | - | FKQ53_RS15620 | 369 | IS3 family transposase |
| 4052886 | 4054120 | + | FKQ53_RS17875 | 411 | IS3 family transposase |
| 4068661 | 4070199 | - | FKQ53_RS17955 | 512 | IS3 family transposase |
| 4070253 | 4071382 | - | FKQ53_RS17960 | 376 | IS3 family transposase |
| 4088415 | 4088723 | + | FKQ53_RS18030 | 102 | transposase |
| 4088720 | 4089565 | + | FKQ53_RS18035 | 281 | IS3 family transposase |
| 4089656 | 4091194 | + | FKQ53_RS18040 | 512 | IS3 family transposase |
| 4403911 | 4405161 | - | FKQ53_RS19435 | 416 | IS256 family transposase |
| 4919267 | 4920501 | - | FKQ53_RS21730 | 411 | IS3 family transposase |
| 4924191 | 4925207 | - | FKQ53_RS21760 | 338 | IS30 family transposase |
| 4933791 | 4934831 | - | FKQ53_RS21825 | 346 | tyrosine-type recombinase/integrase |
| 4980424 | 4981125 | - | FKQ53_RS22070 | 233 | MULTISPECIES: transposase |


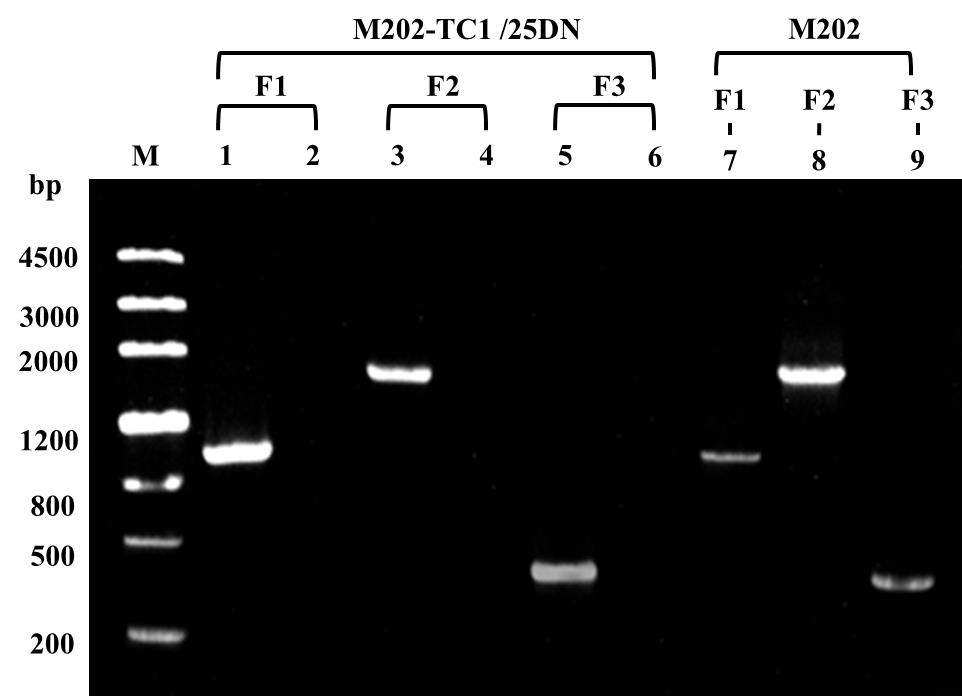


**Figure S1.** Verification of GI-M202a by PCR. M, DNA marker. F1-F3 fragments were amplified by primer pairs V1-F/R, V2-F/R and V3-F/R, respectively. The following templates were used in PCR: Lanes 1, 3, and 5, total DNA fragments extracted from conjugant M202-TC1; Lanes 2, 4, and 6, total DNA fragments extracted from 25DN; and Lanes 7-9, total DNA fragments extracted from M202. Full-length gel is presented in Supplementary Figure S3.


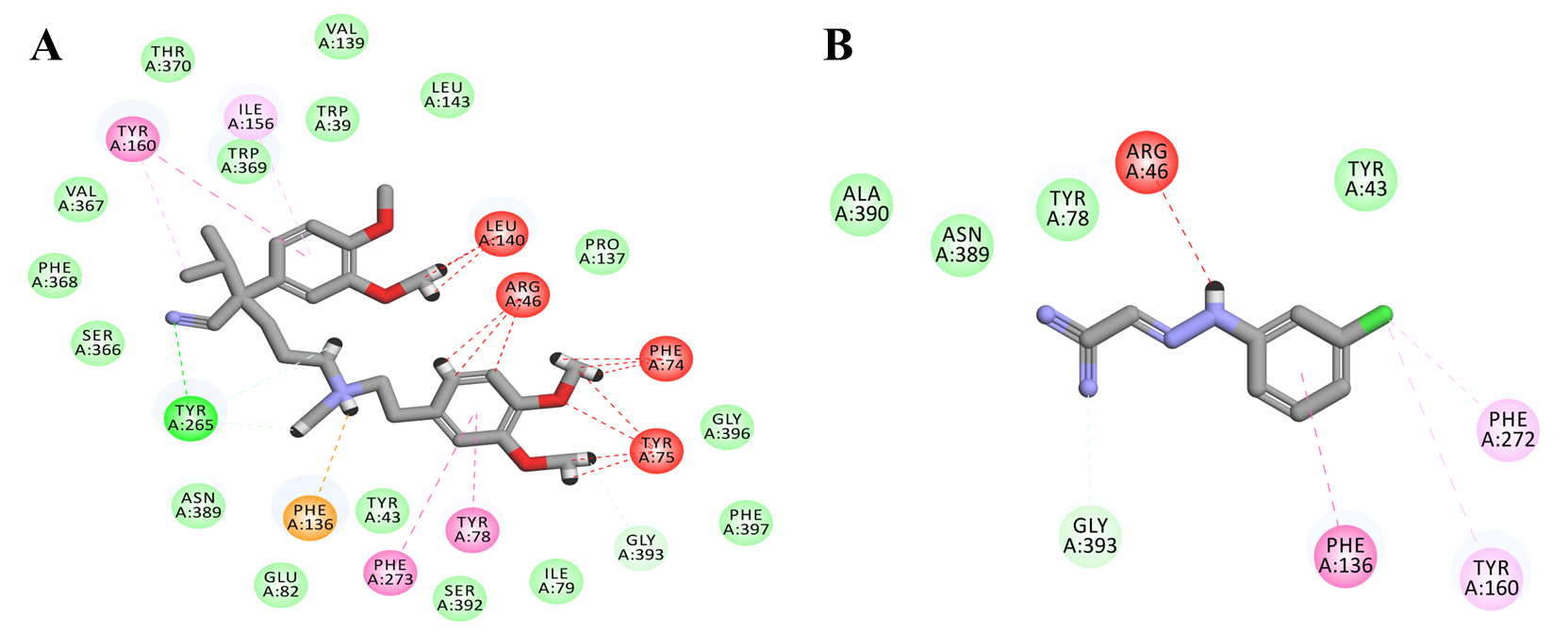


**Figure S2.** Binding amino acids of inhibitors with FKQ53_RS21695. (A) Interactions of verapamil with FKQ53_RS21695. (B) Interactions of CCCP with FKQ53_RS21695.


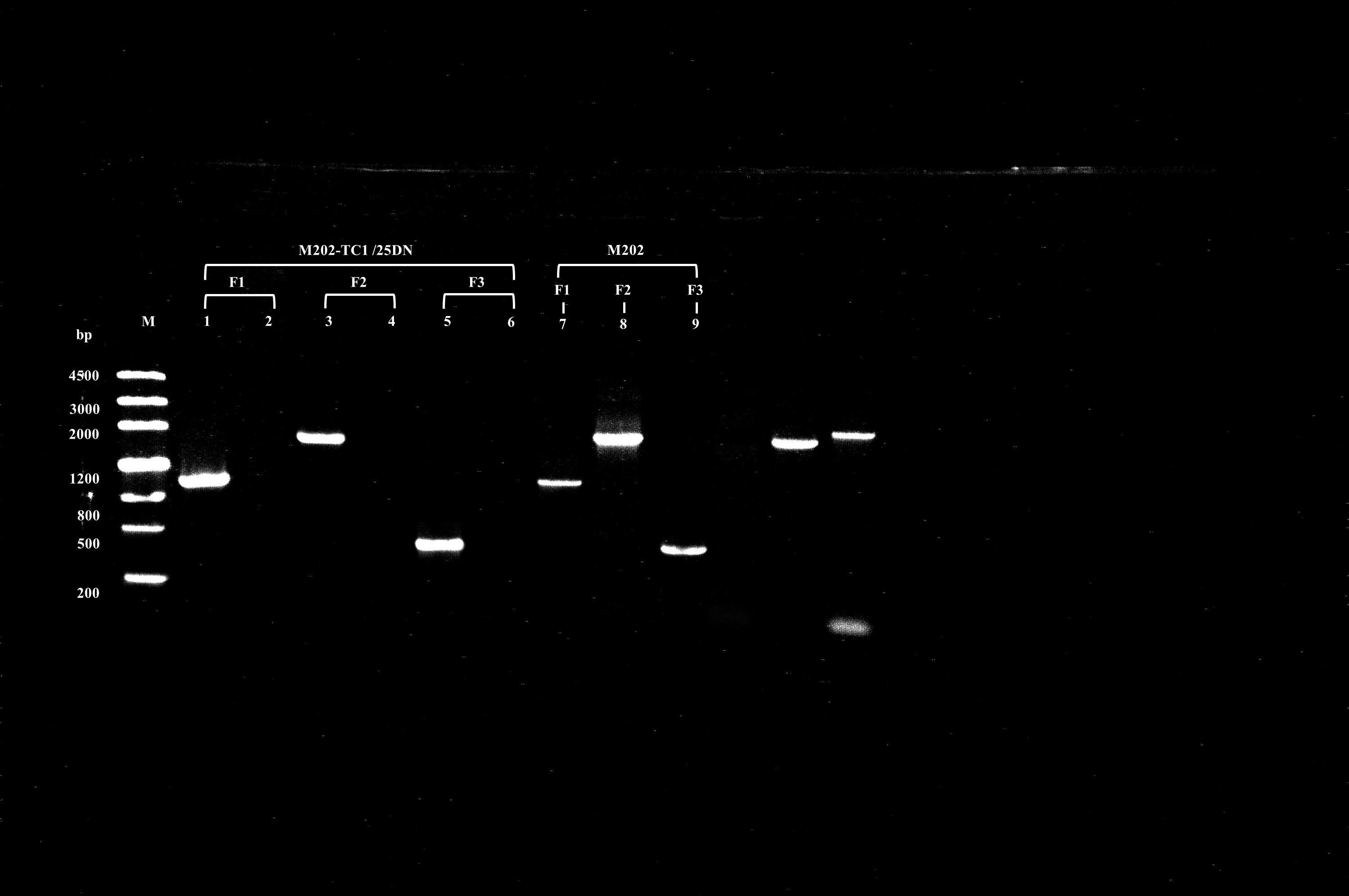


**Figure S3.** Full-length gel of Figure 3. Figure 3 in manuscript was cropped from lane1 to lane 9, lane 10 to lane 12 were cropped out.
